# Identification of a novel prognostic signature correlated with epithelial‐mesenchymal transition, N6‐methyladenosine modification, and immune infiltration in colorectal cancer

**DOI:** 10.1002/cam4.5384

**Published:** 2022-10-25

**Authors:** Xiao Qu, Honghong Tan, Jingxian Mao, Mengxue Yang, Jian Xu, Xuebing Yan, Wenjuan Wu

**Affiliations:** ^1^ Department of Gastrointestinal Surgery, Shanghai Tenth People's Hospital Tongji University School of Medicine Shanghai China; ^2^ Department of VIP Clinic, General Division, Shanghai East Hospital Tongji University School of Medicine Shanghai China; ^3^ Department of Gastroenterology Shanghai East Hospital Ji'an Hospital Ji'an China; ^4^ Department of Oncology The Affiliated Hospital of Yangzhou University, Yangzhou University Yangzhou China; ^5^ Department of Oncology Northern Jiangsu People's Hospital affiliated to Yangzhou University, Yangzhou University Yangzhou China

**Keywords:** colorectal cancer, epithelial‐mesenchymal transition, immunity and biomarker, N6‐methyladenosine modification

## Abstract

**Objective:**

Colorectal cancer (CRC) is a commonly diagnosed human malignancy worldwide. Both epithelial‐mesenchymal transition (EMT) and N6‐methyladenosine (m6A) modification play a crucial role in CRC development. This study aimed to construct a prognostic signature based on the genes related to EMT and m6A modification.

**Method:**

Firstly, the mRNA expression profiling of CRC tissues was analyzed using TCGA and GEO databases. The prognostic hub genes related to EMT and m6A modification were selected using weighted correlation network and cox regression analysis. The prognostic signature was constructed based on hub genes, followed by validation in three external cohorts. Finally, the expression of the representative hub gene was detected in clinical samples, and its biological role was investigated using assays in vivo and in vitro.

**Results:**

A prognostic signature was constructed using the following genes: YAP1, FAM3C, NUBPL, GLO1, JARID2, NFKB1, CDKN1B, HOOK1, and GIPC2. The signature effectively stratified the clinical outcome of CRC patients in the training cohort and two validation cohorts. The subgroup analysis demonstrated the signature could identify high‐risk population from CRC patients within stage I‐II or III‐IV, female, male and elder patients. The signature was correlated with the infiltration of some immune cells (such as macrophage and regulatory T cells) and gene mutation counts. Finally, the hub gene GIPC2 was found to be downregulated in CRC tissues and most CRC cells lines. GIPC2 overexpression inhibited the malignant characteristics of CRC cells in vitro and in vivo through upregulating E‐cadherin and downregulating N‐cadherin, Vimentin, and Snail, while the opposite results were observed for GIPC2 knockdown in CRC cells.

**Conclusion:**

Our present study for the first time constructed a novel prognostic signature related to EMT, m6A modification, and immune infiltration for CRC risk stratification. In addition, GIPC2 is identified as a promising clinical biomarker or therapeutical target for CRC.

## Graphical abstract

Identification of a novel prognostic signature for colorectal cancer risk stratification.

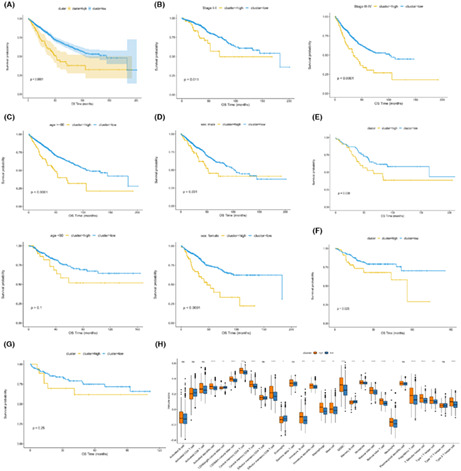

## 1 INTRODUCTION

Colorectal cancer (CRC) is a commonly diagnosed human malignancy worldwide, ranking the third in cancer mortality.^1^ According to the GLOBOCAN report, the estimated number of new CRC cases will increase to 3,154,674 in 2040 at an alarming speed.^2^ Despite of improved screening in high‐risk population and multi‐disciplinary therapy, a considerable proportion of CRC patients suffer from postoperative recurrence and anti‐cancer drug resistance, suggesting current management of CRC fails to arrive at a satisfactory level.^3^ It is well‐established that CRC development is complicated involving various molecular events, implying the potential application of molecular biomarkers in CRC diagnosis and therapy. In addition to traditional biomarkers such as Ki‐67, KRAS, and BRAF, some novel biomarkers such as β‐catenin, CXCR4, SDF‐1, and TGF‐β are being tested in clinical or preclinical studies but their actual applications seem to be far from ideal.^4^ Therefore, it is an urgent and challenging task to identify and validate reliable molecular biomarkers for CRC management.

Epithelial‐mesenchymal transition (EMT), defined as a biological process where epithelial cells acquire mesenchymal phenotype, is a key molecular event mediating tumor metastasis and therapy resistance in CRC.^5^ At the molecular level, EMT is commonly triggered by transcription factors including Snail, ZEB1/2, and Twist1/2, resulting in downregulation of epithelial markers (such as E‐cadherin) and upregulation of mesenchymal markers (such as N‐cadherin and vimentin). There is a growing body of evidence indicating the prognostic value of these EMT‐related proteins in CRC patients. For instance, a recent study had demonstrated expression of E‐cadherin in liver metastases and expression of vimentin in serum serve as indicators for early recurrence after liver resection in metastatic CRC patients.^6^ A meta‐analysis including 16 retrospective studies has linked the expressions of EMT transcription factors with poor overall survival (OS) and distant metastasis in CRC patients.^7^ Furthermore, several bioinformatics‐based researches have developed and validated EMT signatures for identifying high‐risk CRC patients suffering poor prognosis.^8^, ^9^ In addition to EMT, emerging studies have suggested N6‐methyladenosine (m6A) modification also serves as a crucial molecular mechanism underlying CRC development.^10^ The m6A modification, known as the most abundant RNA modification in eukaryote, is induced by writers (METTL3, METTL14, etc.), removed by erasers (FTO, ALKBH5, etc.), and recognized by readers (YTHDF1/2/3, IGF2BP, etc.). Recent evidences have closely linked m6A modification with EMT in CRC development. METTL14‐mediated m6A modification is identified by YTHDF2 and therefore promotes the mRNA degradation of SOX4, inhibiting the EMT process of CRC cells.^11^ METTL3‐mediated m6A modification upregulates the expression of circRNA‐1662, subsequently accelerates YAP1 nuclear localization, and finally promotes EMT‐induced CRC metastasis.^12^ Considering the crucial role of both the molecular events and their correlation in CRC, identification of genes related to them may provide novel clinical indicators or therapeutical targets for CRC management.

In this study, the mRNA expression profiling of The Cancer Genome Atlas (TCGA) and Gene Expression Omnibus (GEO) database was firstly utilized to select hub genes related to EMT and m6A modification. Then, a prognostic signature was constructed based on the selected genes and validated in three independent cohorts. Meanwhile, the correlation between the signature and immune infiltration was investigated. Finally, the expression of the representative hub gene was detected in clinical samples and CRC cell lines, followed by biological function validations in vitro and in vivo. The present work will provide novel insights into the clinical application of EMT and m6A modification and contribute to the biomarker‐directed precise medicine.

## 2 MATERIALS AND METHODS

### 2.1 Patient information

For constructing the prognostic signature in the test cohort, the mRNA expression profiling of 616 and 566 CRC tissues was, respectively, downloaded from TCGA database (https://portal.gdc.cancer.gov/) and GEO database (https://www.ncbi.nlm.nih.gov/geo/, GSE39582 cohort). For validating the signature efficacy, the following three GEO cohorts were employed: GSE103479 (*n* = 156), GSE14333 (*n* = 226), and GSE38832 (*n* = 122). In addition, the data of 1184 EMT genes were downloaded from dbEMT database (http://www.dbemt.bioinfo‐minzhao.org). For detecting the expression of the hub gene in tumor tissues, 40 pairs of CRC tissues and matched adjacent normal tissues were collected from CRC patients receiving radical surgery between December 2021 and March 2022, at the Department of General Surgery, Affiliated Hospital of Yangzhou University. None of the patients were diagnosed at IV stage and received neoadjuvant chemoradiotherapy. This study was approved by the ethics committees of Affiliated Hospital of Yangzhou University (No. 2020‐YKL04‐Y011). Written informed consents were obtained from patients for using their tissues and clinical information in medical researches.

### 2.2 Bioinformatics analysis

The mRNA expression profiling of TCGA and GSE39582 cohort was integrated using the ComBat function of R project. The cluster analysis was performed to stratify the entire cohort into three clusters based on the following 21 m6A modification regulators: “METTL3,” “METTL14,” “RBM15,” “RBM15B,” “WTAP,” “KIAA1429,” “CBLL1,” “ZC3H13,” “ALKBH5,” “FTO,” “YTHDC1,” “YTHDC2,” “YTHDF1,” “YTHDF2,” “YTHDF3,” “IGF2BP1,” “HNRNPA2B1,” “HNRNPC,” “FMR1,” “LRPPRC,” and “ELAVL1”. Then, the expression data of EMT genes were analyzed using Weighted correlation network analysis (WGCNA), followed by selection of the key module related to m6A modification. The cox regression analysis was used to determine the candidate hub genes significantly affecting the OS of CRC patients (*p* < 0.05). The signature was constructed based on the hub genes, and the risk score was calculated using the principal component analysis. The high‐risk group was classified according to the median value of the risk scores. For selecting GEO cohorts in the validating phase, the following inclusion criteria were used: (1) the cohorts only including colon and/or rectal cancer patients; (2) the cohorts with complete patient information including clinicopathological parameters and follow‐up records; (3) the cohorts with the available mRNA expression values for all the selected hub genes. The proportion of infiltrated immune cells was estimated using single sample gene set enrichment analysis. The copy number variation (CNV) was analyzed using GenePattern (https://cloud.genepattern.org/gp/pages/index.jsf).

### 2.3 Cell culture

The human CRC cell lines (SW620, HCT‐116, SW480, HT‐29, and LoVo) and normal intestinal epithelial cell line (NCM460) were purchased from the Type Culture Collection of the Chinese Academy of Sciences and the INCELL Corporation, respectively. The key molecular backgrounds of CRC cells were shown in Table S1. The cells were maintained in the following culture mediums supplemented with 10% fetal bovine serum (Gibco) at 37°C in a humidified atmosphere of 5% CO2: McCoy'5a culture medium (HCT‐116, HT‐29, and NCM460), L‐15 culture medium (SW620 and SW480), and F12K culture medium (LoVo).

### 2.4 Short hairpin RNA and plasmid construction

Since we found GIPC2 was significantly downregulated in CRC tissues and poorly investigated in CRC field, we next aimed to clarify its biological role in CRC development. The procedures of short hairpin RNA (shRNA)/plasmid construction and lentivirus transfection were described in our previous work.^13^ For stable knockdown (KD) of GIPC2, the sequences of the shRNA and negative control (NC) were designed as follows: GIPC2‐shRNA, 5'‐GGAAGAAATGCCTTCTGAAAC‐3′; NC, 5'‐CCTAAGGTTAAGTCGCCCTCG‐3′. The DNA oligos containing shRNA sequence were synthesized and cloned into vectors, followed by DNA sequencing. The lentivirus (GENERAY Biotechnology) was synthesized using the vectors and then transfected into HEK‐293 T cells. The lentivirus supernatant was collected and purified after 48 h incubation. The CRC cells with stable GIPC2 knockdown were screened using puromycin (Sigma‐Aldrich). For stable GIPC2 overexpression, the sequence of the plasmid was designed using the NCBI reference sequence. The plasmid was cloned into the vector, and the CRC cells were infected with the lentivirus carrying the vector. The knockdown and overexpression efficacy were validated using Western blot.

### 2.5 Quantitative real‐time PCR (qRT‐PCR)

Total RNA was extracted from tissues using TRIzol reagent (Invitrogen) according to the manufacturer's instructions. The obtained RNA was reverse‐transcribed into cDNA, and the reaction was conducted using SYBR Green PCR Kit (Takara Biotechnology). The primer sequences of the genes and reaction conditions were provided in Tables S2 and S3, respectively. The *GAPDH* gene functions as an internal control gene and the relative mRNA levels of genes were calculated using the 2^−ΔΔCT^ method.

### 2.6 Western blot

The total protein was extracted from cells and tissues using the lysis buffer (Beyotime Biotechnology). The concentration of the obtained protein was measured using the bicinchonininc acid (BCA) protein assay kit (Thermo Fisher Scientific). The protein samples were loaded onto a 10% SDS–polyacrylamide gel and transferred to a polyvinylidene difluoride membrane. After incubation with 5% skimmed milk for 2 h, the membrane was incubated with following primary antibodies overnight at 4°C: anti‐GIPC2 (Abcam, ab175272, 1:1000), anti‐E‐cadherin (Cell Signaling Technology, 14,472, 1:1000), anti‐N‐cadherin (Cell Signaling Technology, 13,116, 1:1000), anti‐Snail (Cell Signaling Technology, 3879, 1:1000), anti‐Vimentin (Cell Signaling Technology, 5741, 1:1000), anti‐β‐actin (Cell Signaling Technology, 3700, 1:5000), and anti‐GAPDH (Cell Signaling Technology, 5174, 1:5000). Then, the membrane was incubated with a secondary antibody (Cell Signaling Technology, 7074, 1:5000) for 1.5 h at room temperature. Finally, the blot visualization was carried out using a Chemiluminescence Detection Kit (Thermo Fisher Scientific). GAPDH and β‐actin function as internal control proteins.

### 2.7 Immunohistochemistry

The paraffin‐embedded tissues were cut into 5 μm thick sections, dewaxed in xylene, and rehydrated in ethanol with gradient concentrations. Antigen retrieval was performed through microwave heating, and endogenous peroxidase activity was blocked using 3% hydrogen peroxide solution. Then, the sections were incubated with the primary antibody against Ki‐67 (Cell Signaling Technology, 62,548, 1:100) overnight at 4°C. Finally, the sections were incubated with a secondary antibody (Cell Signaling Technology, 8125, 1:200) for 1 h at 37°C. The protein staining was visualized using diaminobenzidine tetrahydrochloride reagent (Invitrogen). For evaluation, the positive staining of Ki‐67 was located in the nucleus of CRC cells and the percentages of positive cells in three random fields in each section were recorded.

### 2.8 Cell counting kit‐8 assay

The CRC cells were seeded into 96‐well culture plates with 100 μl per well. After overnight incubation, each well was supplemented with 10 μl of Cell Counting Kit‐8 (CCK‐8) reagent (Solarbio Life Sciences). After 4 h incubation, the optical density (OD) value of each well was measured using a microplate reader (Thermo Fisher Scientific) at 450 nm wavelength.

### 2.9 Colony formation assay

The CRC cells were seeded into 6‐well culture plates with 200 cells per well. After incubation in a 37°C and 5% CO2 cell incubator for 14 days, the colonies were fixed with 4% paraformaldehyde for 15 min and stained with crystal violet for 25 min. Finally, the stained colonies were counted under a microscopy.

### 2.10 Transwell invasion and migration assay

A total of 4 × 10^4^ CRC cells were seeded into the upper chamber containing the serum‐free medium. For invasion assay, a matrigel was additionally added into the bottom of the up chamber before cell seeding. The lower chamber was supplemented with the complete culture medium. After 24 h incubation, the cells in the lower chamber were fixed with methanol and stained with crystal violet for 40 min. The stained cells of five random fields were counted under a microscopy.

### 2.11 Xenograft model

A total of 24 male BALB/c nude mice (4 weeks old, 16‐18 g) were purchased from Shanghai Slack Laboratory Animals Co., Ltd, Shanghai, China. 6 × 10^6^ CRC cells were injected subcutaneously into the right hind flank of each mouse. Tumor volumes were measured every two days using a vernier caliper, and the volume was calculated as 0.5 × length × width.^2^ Three weeks after injection, the mice were sacrificed and the harvested tumors were embedded in paraffin. This study was approved by the ethics committees of Affiliated Hospital of Yangzhou University (No. 2020‐YKL04‐Y011).

### 2.12 Statistical analysis

The data are presented as mean ± standard deviation (SD). The statistical analysis was performed using SPSS 22.0 software or GraphPad Prism 8 software. The bilateral Student's *t* tests were used to compare the difference between the two groups. The Kaplan–Meier model was used to depict survival curves, and the survival difference was compared by the log‐rank test. The proportions of infiltrated immune cells between the two groups were compared using the Wilcoxon test. A p value less than 0.05 was considered statistically significant.

## 3 RESULTS

### 3.1 Identification of hub genes related to EMT and m6A modification

**FIGURE 1:**
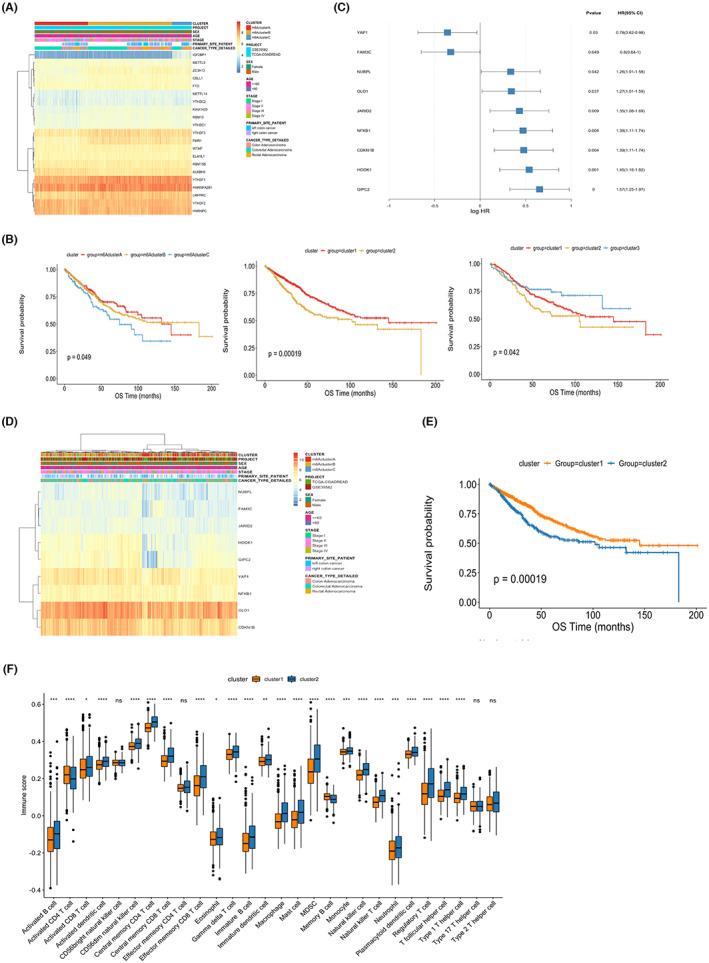
Identification of hub genes related to EMT and m6A modification. (A) Correlations of m6A modification regulators with clinical features in the test cohort (TCGA+GSE39582). (B) The m6A modification phenotype stratifies the survival of CRC patients in the test cohort (left), TCGA (middle), and GSE39582 (right). (C) The Cox regression analysis identifies the nine hub genes significantly correlated with patient prognosis. (D) Correlations of the nine hub genes with clinical features in the test cohort. (E) The molecular phenotype based on the hub genes stratifies the survival of CRC patients in the test cohort. (F) Correlations of the molecular phenotype with immune infiltration in the test cohort.

Firstly, we analyzed the expressions of 21 m6A modification regulators in the test cohort (TCGA+GSE39582), and their correlations with the clinical features of CRC patients were shown in Figure 1A. The cluster analysis based on the K‐means method was then used to stratify the test cohort into three subgroups. The survival analysis demonstrated this m6A modification molecular classification significantly stratified the survival of CRC patients in the test cohort (Figure 1B, left). The similar results were also observed in the subgroup analysis stratified by TCGA and GSE39582 cohort (Figure 1B, middle and right). Then, based on the 1184 EMT genes, the WGCNA was utilized to select the key module related to m6A modification. As a result, 75 candidate genes were selected and the Cox regression analysis further identified 9 hub genes related to the survival of CRC patients (Figure 1C). The correlation of these hub genes with the clinical features of CRC patients was shown in Figure 1D. Based on the expression data of 9 hub genes, the cluster analysis stratified the test cohort into two clusters and there was a significant survival difference between the clusters (Figure 1E). The function and pathway analysis demonstrated differentially expressed genes between the clusters were enriched in cell adhesion molecule binding, PI3K‐AKT signaling pathway, and so on (Figure S1). Moreover, there was a significant difference of the proportions of most immune cells between the clusters, such as B‐cell, T‐cell, and natural killer cell (Figure 1F). Finally, the CNV analysis demonstrated there was no significant difference of the mutation counts between the clusters (Figure S2).

### 3.2 Construction and validation of the signature related to EMT, m6A modification, and immune infiltration

**FIGURE 2:**
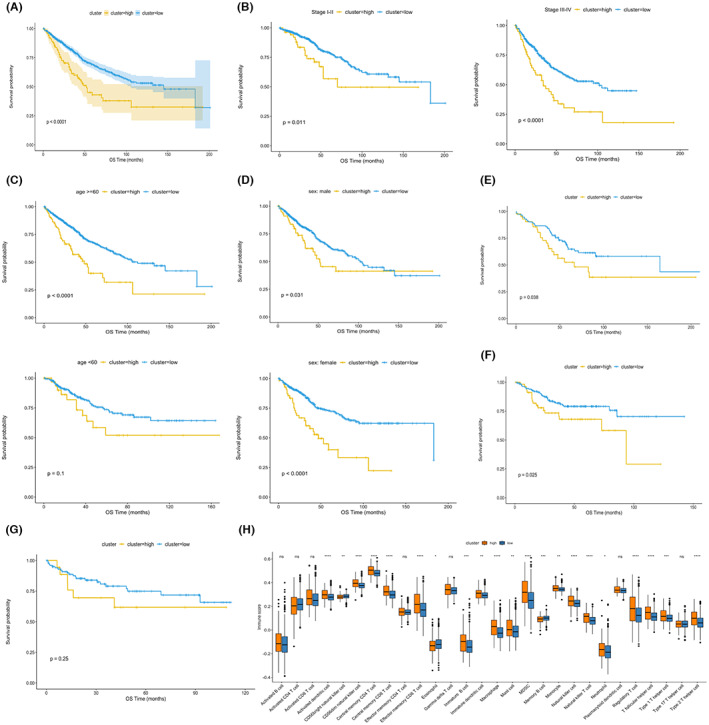
Construction and validation of the signature related to EMT, m6A modification, and immune infiltration. (A) The signature based on the nine hub genes stratifies the survival of CRC patients in the test cohort. (B) The performance of the signature is validated in CRC patients within stage I‐II (left) or III‐IV (right). (C) The signature stratifies the survival of CRC patients aged over 60 years (upper) instead of those younger than 60 years (lower). (D) The performance of the signature is validated in male (upper) or female CRC patients (lower). (E‐F) The performance of the signature is validated in CRC patients from GSE103479 (E) and GSE14333 (F). (G) The signature fails to stratify the survival of CRC patients from GSE38832. (H) Correlations of the signature with immune infiltration in the test cohort.

For better utilization of the nine hub genes in prognosis prediction, they were then integrated into a signature that stratified the test cohort into high‐ and low‐risk groups. As shown in Figure 2A, the high‐risk group had a significantly worse survival than the low‐risk group. For clarifying its efficacy in patients with the same clinical feature, the subgroup analysis was performed based on TNM stage, age, and gender. The result demonstrated the signature could effectively stratify the survival of both the patients within I‐II and III‐IV stage (Figure 2B). Similar results were also observed in patients aged over 60 years, male and female patients (Figure 2C,D). However, although we did observe the better survival tendency in the low‐risk group, the difference was not statistically significant in patients younger than 60 years old (Figure 2C). Moreover, three validation cohorts from GEO database were utilized to confirm the prognostic value of the signature. As a result, the signature was found to effectively stratify the survival of CRC patients in GSE103479 (Figure 2E) and GSE14333 (Figure 2F) cohort, but failed in GSE38832 cohort (Figure 2G). The proportions of infiltrated immune cells were compared between the high‐ and low‐risk groups (Figure 2H). The result demonstrated the proportions of some tumor‐related immune cells (such as macrophage and regulatory T cells) were significantly higher in the high‐risk group as compared with the low‐risk group. Finally, the CNV analysis demonstrated the gene mutation count of the high‐risk group was dramatically more than that of the low‐risk group (Figure S3).

### 3.3 Expression of GIPC2 in CRC tissues and cell lines

**FIGURE 3:**
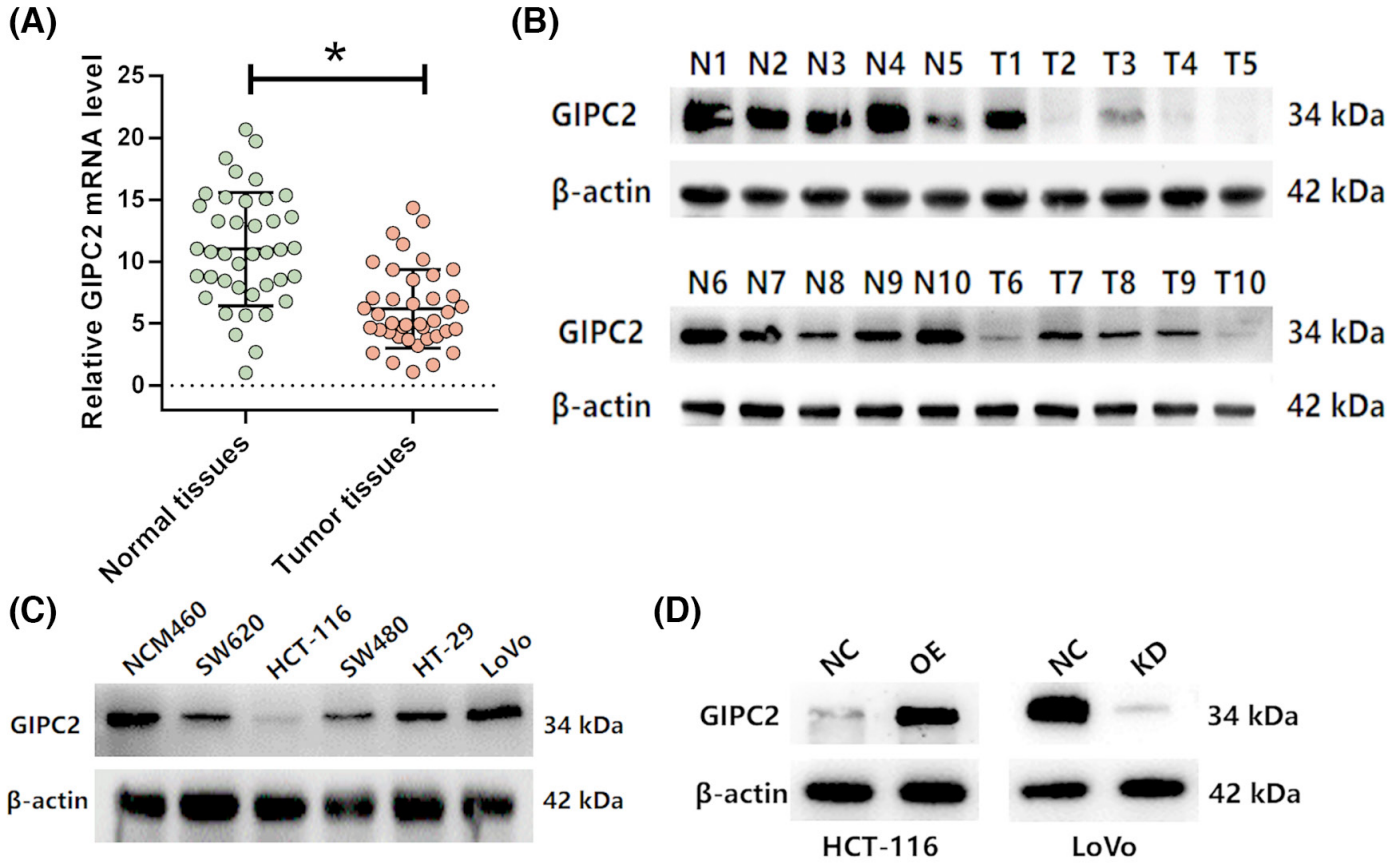
Expression of GIPC2 in CRC tissues and cell lines. (A) The qRT‐PCR detects the mRNA expression of GIPC2 in CRC and adjacent normal tissues. (B) The Western blot detects the protein expression of GIPC2 in CRC and adjacent normal tissues. (C) The Western blot detects the protein expression of GIPC2 in five CRC cell lines and one normal intestinal epithelial cell line (NCM460). (D) The Western blot confirmed the efficacy of GIPC2 overexpression and knockdown in HCT‐116 (left) and LoVo cells (right).

Firstly, we used qRT‐PCR to detect the mRNA expression of the nine hub genes in 40 pairs of CRC tissues and matched adjacent normal tissues (Figure 3A and Figure S4). The result demonstrated the mRNA levels of GIPC2 and HOOK1 were significantly lower in the CRC tissues than that in matched normal adjacent normal tissues, while it was opposite for GLO1, NFKB1, and YAP1. Since GIPC2 was rarely investigated in solid tumors and had the highest HR value in the survival analysis, therefore we focused on it in our following biological assays. Then, the Western bolt confirmed that the protein level of GIPC2 was also lower in the CRC tissues than that in matched normal adjacent normal tissues (Figure 3B). The Western blot was also used to detect the protein expression of GIPC2 in one normal intestinal epithelial cell line (NCM460) and five CRC cell lines (SW620, HCT‐116, SW480, HT‐29, and LoVo). The result demonstrated the protein expression of GIPC2 was significantly higher in the NCM460 cells as compared with SW620, HCT‐116, SW480, and HT‐29 cells (Figure 3C). In addition, HCT‐116 and LoVo had the lowest and highest protein expression of GIPC2, respectively, among the CRC cell lines. Therefore, the two cell lines were selected for the following functional assays. The Western blot confirmed the efficacy of GIPC2 overexpression and knockdown in HCT‐116 and LoVo cells, respectively (Figure 3D).

### 3.4 GIPC2 inhibits the malignant characteristics of CRC cells through regulating EMT

**FIGURE 4:**
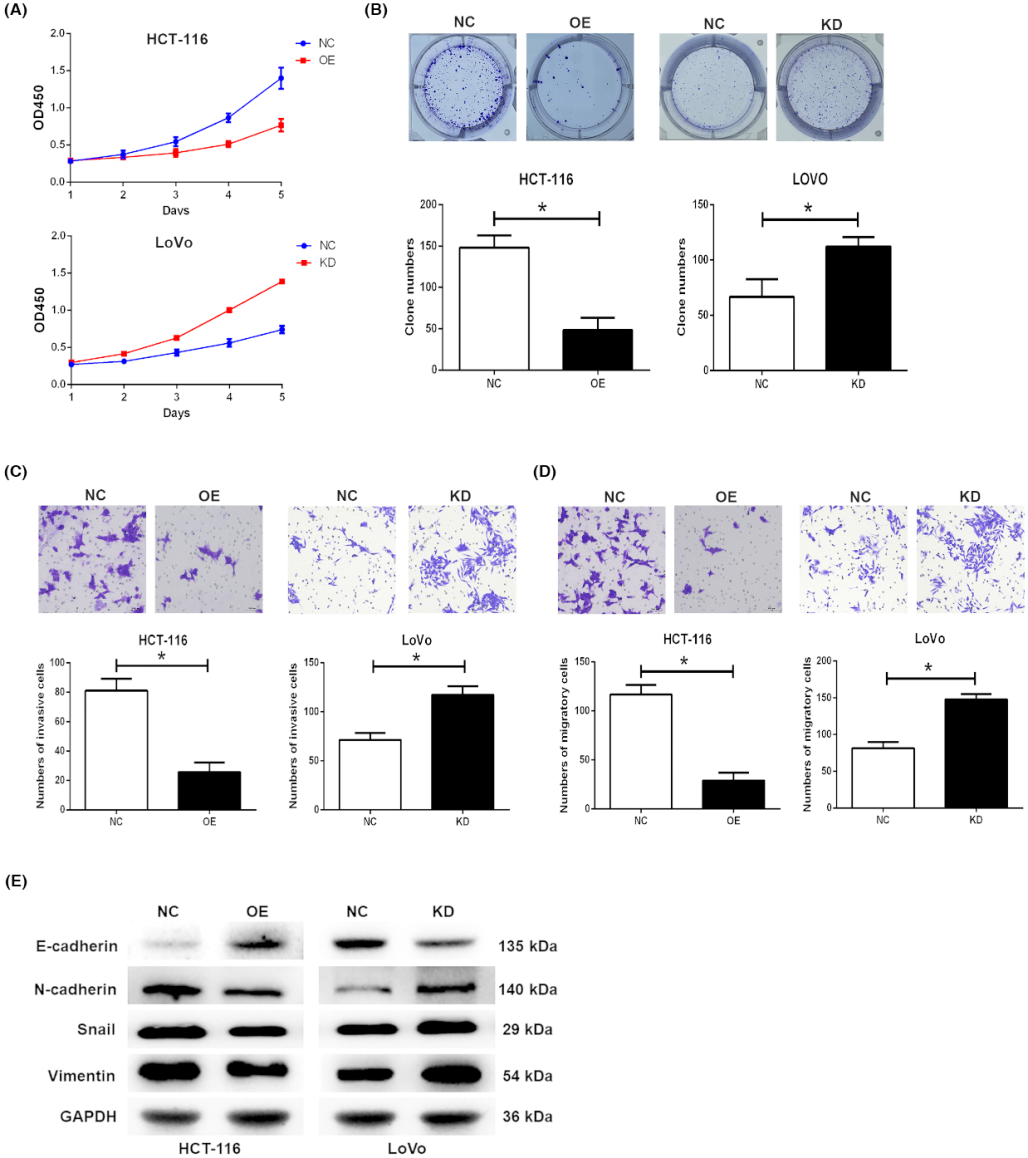
GIPC2 inhibits the malignant characteristics of CRC cells through regulating EMT. (A) GIPC2 overexpression inhibits the proliferation of HCT‐116 cells (upper), and GIPC2 knockdown exerts the opposite effect in LoVo cells (lower). (B) GIPC2 overexpression reduces the colony number of HCT‐116 cells (left), and GIPC2 knockdown exerts the opposite effect in LoVo cells (right). (C‐D) GIPC2 overexpression inhibits the invasion (C) and migration (D) of HCT‐116 cells (left), and GIPC2 knockdown exerts the opposite effect in LoVo cells (right). (E) GIPC2 overexpression upregulates E‐cadherin expression but downregulates N‐cadherin, Vimentin, and Snail expression in HCT‐116 cells (left), and GIPC2 knockdown exerts the opposite effect in LoVo cells (right).

The CCK‐8 assay was firstly performed to investigate the role of GIPC2 in the proliferation of CRC cells. GIPC2 overexpression effectively inhibited the proliferative ability of HCT‐116 cells, while the opposite was observed in GIPC2 knockdown in LoVo cells (Figure 4A). In the colony formation assay, GIPC2 overexpression significantly decreased the colony number of HCT‐116 cells, while the opposite was observed in GIPC2 knockdown in LoVo cells (Figure 4B). In the invasion assay, GIPC2 overexpression significantly inhibited the invasive ability of HCT‐116 cells, while GIPC2 knockdown exerted the opposite effect in LoVo cells (Figure 4C). Similar results were also observed in the migration assay (Figure 4D). Finally, Western blot was used to detect the protein expression of EMT markers in CRC cells after GIPC2 overexpression or knockdown (Figure 4E). The result showed GIPC2 overexpression upregulated E‐cadherin expression, but decreased the N‐cadherin, Vimentin, and Snail expression in HCT‐116 cells. The opposite result was observed for GIPC2 knockdown in LoVo cells.

### 3.5 GIPC2 inhibits the growth of CRC cells in vivo

**FIGURE 5:**
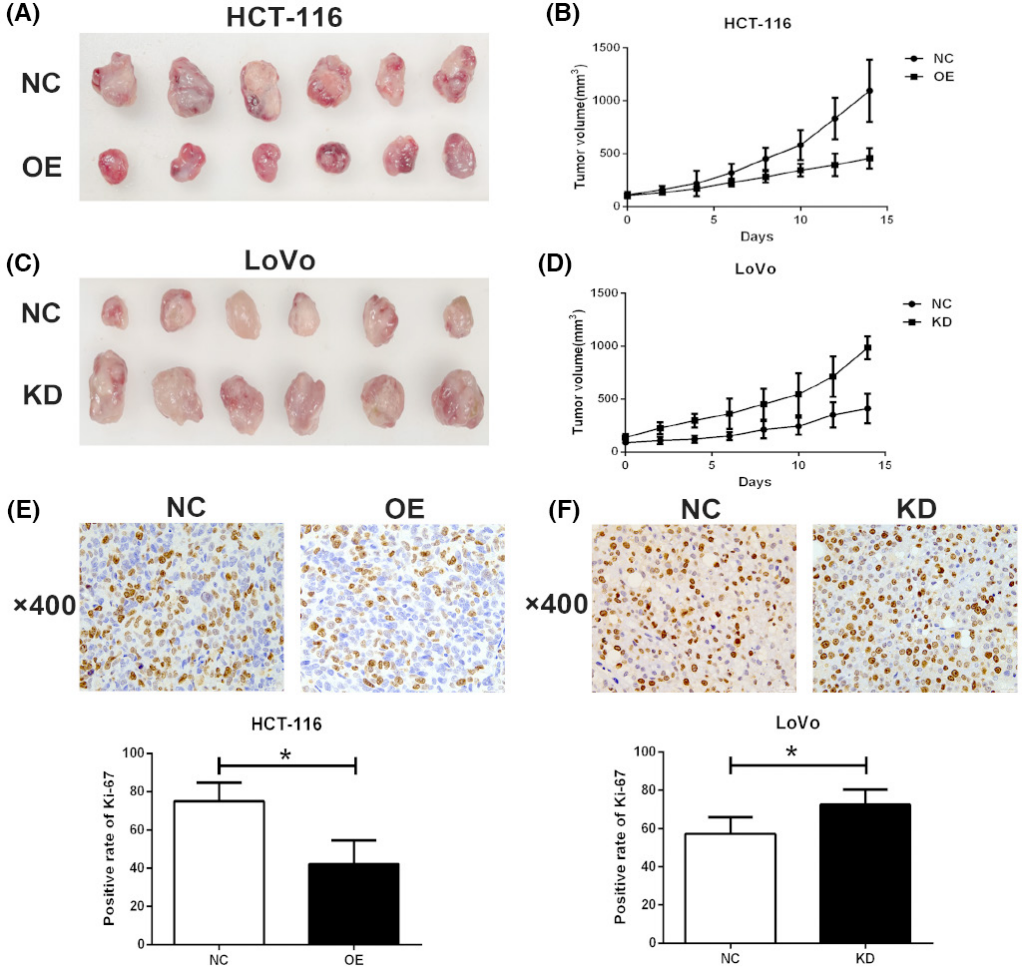
GIPC2 inhibits the growth of CRC cells in vivo. (A) Harvested xenografts of HCT‐116 cells. (B) GIPC2 overexpression inhibits the growth of the xenografts from HCT‐116 cells. (C) Harvested xenografts of LoVo cells. (D) GIPC2 knockdown promotes the growth of the xenografts from LoVo cells. (E) GIPC2 overexpression reduces the positive rate of Ki‐67 in the xenografts of HCT‐116 cells. (F) GIPC2 knockdown increases the positive rate of Ki‐67 in the xenografts of LoVo cells.

To investigate the biological role of GIPC2 in vivo, a xenograft model was established by injecting CRC cells subcutaneously into the right hind flank of nude mice. The harvested xenografts of HCT‐116 and LoVo cells were shown in Figure 5A,C, respectively. The growth curves demonstrated GIPC2 overexpression significantly slowed down the growth speed of the xenografts derived from HCT‐116 cells (Figure 5B), while GIPC2 knockdown exerted the opposite impact on that of the xenografts derived from LoVo cells (Figure 5D). In addition, the immunohistochemistry was used to detect the expression of Ki‐67 in the harvested xenografts. The result demonstrated GIPC2 overexpression reduced the positive rate of Ki‐67 in the xenografts derived from HCT‐116 cells (Figure 5E), while the opposite was observed in the xenografts derived from LoVo cells with GIPC2 knockdown (Figure 5F).

## 4 DISCUSSION

With the rapid development of medical technology, the clinical management of CRC patients has become more individualized than before, including multi‐disciplinary therapies, biomarker detection, and risk stratification. Due to high tumor heterogeneity, it is challenging for clinicians to accurately identify CRC patients with high recurrence or progression risk merely based on clinical clinicopathological features, suggesting the crucial role of molecular biomarkers.^14^, ^15^, ^16^, ^17^, ^18^ EMT is a classical molecular mechanism regulating CRC progression and its markers as well as related non‐coding RNAs are identified as reliable predictors for clinical outcome.^5^ The m6A modification is a novel molecular mechanism participating in CRC development through regulating the various RNA processes such as splicing, translation, and degradation.^19^ There is a growing body of studies to establish the prognostic signature based the genes related to EMT or m6A modification.^9^, ^20^, ^21^, ^22^ In the present study, our findings will not only further highlight the clinical application of EMT and m6A modification, but also provide novel drug targets for individualized treatment.

In this study, we established the prognostic signature based on the following hub genes: YAP1, FAM3C, NUBPL, GLO1, JARID2, NFKB1, CDKN1B, HOOK1, and GIPC2. Previous evidences have linked several of these genes with EMT and/or m6A modification in CRC. For instance, increased FAM3C copy numbers were correlated with extramural invasion of CRC, and the mechanism investigation revealed FAM3C promoted cancer cell invasion through E‐cadherin transcription.^23^ NUBPL was found to activate ERK signaling to induce EMT in CRC cells.^24^ LINC021 exerts its oncogenic role in CRC through enhancing the mRNA stability of JARID2 via m6A modification.^25^ CDKN1B serves as a target gene of miR‐200 Family, which have been identified as curial regulators of EMT in CRC.^26^ On the contrary, we noted the role of GLO1, HOOK1, and GIPC2 was rarely investigated in CRC but well identified in other cancers.^27^, ^28^, ^29^ Therefore, more attention should be paid to the clinical significance and biological functions of these genes in CRC.

The constructed signature was found to effectively stratify the OS of patients in the test cohort, which was subsequently confirmed in two external cohorts (GSE103479 and GSE14333). However, it is worth mentioning that the signature failed to significantly stratify the OS of CRC patients in GSE38832, suggesting the necessity of more validations in future. In addition, the subgroup analysis revealed the signature was able to identify high‐risk subpopulation from patients with the same clinical features including TNM stage, gender, and age, except for those younger than 60 years old. In this regard, we suggest our signature may contribute to more precise risk stratification in clinical practice. Although aging is an adverse prognostic factor, the CRC incidence of the young is increasing rapidly and many of young CRC patients are initially diagnosed at advanced stage.^30^ In this study, despite no statistical significance, we did observe the high‐risk group had a worse OS than the low‐risk group in patients younger than 60 years. Previous studies have provided several promising molecular biomarkers for outcome prediction in the young CRC patients such as platelet‐derived growth factor receptor α and B‐cell lymphoma 2.^31^, ^32^ Our following work will focus on signature improvement for young CRC patients through integrating these candidate biomarkers or specific clinical features.

In this study, we found the signature was correlated with the proportion changes of some immune cells such as macrophage and regulatory T cell, indicating the potential correlation of immune cells with EMT or m6A modification. A comprehensive analysis of major RNA adenosine modifications (including m6A modification) indicated alterations of modification writers were correlated with M2 macrophages, EMT activation, and oncogenic pathways such as MAPK and EGFR.^33^ The METTL3/METTL14 mediated m6A modification promoted the expression of PSMC5, subsequently resulting in EMT‐induced CRC metastasis and increased infiltration of M2 macrophages and N2 neutrophils.^34^ In addition, some m6A or EMT signatures were found to correlate with immune cell infiltration in CRC, partly supporting our findings.^35^, ^36^, ^37^ Therefore, the predictive role of the signature in cancer immunotherapy needs further investigation. Finally, we found the high‐risk group had more gene mutation counts than the low‐risk group, suggesting mutation test‐directed therapy may be preferentially recommended in these patients.^38^

Since GIPC2 as a hub gene of the signature was poorly investigated in cancers, we next made efforts to clarify its role in CRC. We found GIPC2 was downregulated in CRC tissues and most CRC cell lines, implying its role as a tumor suppressor. The function assays in vivo and in vitro confirmed its inhibitory role in the malignant characteristics of CRC cells. Our finding is consistent with a recent study proving GIPC2 inhibited the tumor growth through regulating p27 transcription.^29^ A bioinformatics analysis demonstrated hypermethylation and low expression of GPIC2 was correlated with worse outcome in papillary renal cell carcinoma.^39^ However, a recent work has found GIPC2 was upregulated in prostate cancer and promoted the tumor metastasis through activating WNT signaling.^40^ A previous review has summarized the biological functions and dysregulation of GIPC family genes in tumors and speculated their oncogenic or tumor suppressive role may be context‐dependent.^41^ Therefore, extensive work is still needed to clarify the role of GIPC2 in tumor initiation and development.

There are several limitations that should be improved in our future work. First of all, our study mainly focused on the biological role of GIPC2 in CRC and more efforts should be made to clarify the specific roles of other hub genes such as HOOK1 and GLO1 in CRC. Secondly, due to the limited follow‐up period, we failed to validate the prognostic value of our signature in our own cohort. In future, we will try our best to collaborate with other hospitals to perform further validations based on sufficient clinical resources. Thirdly, the molecular mechanisms regulated by GIPC2 in inhibiting CRC development are still poorly elucidated and needs further investigations. Finally, more attention should be paid to the complicated interaction among m6A modification, EMT, and tumor immunity.

In summary, our study provides a prognostic signature correlated with EMT, m6A modification, and immune infiltration in CRC. Meanwhile, our study for the first time demonstrates GIPC2 serves as a tumor suppressor through inhibiting EMT in CRC cells. These findings provide novel insights into the molecular mechanisms underlying CRC development, contributing to the discovery of novel biomarkers for precise disease management.

## AUTHOR CONTRIBUTIONS

**Xiao Qu:** Conceptualization (equal); methodology (equal); supervision (equal). **Honghong Tan:** Data curation (equal); formal analysis (equal); writing – original draft (equal). **Jingxian Mao:** Data curation (equal); formal analysis (equal); writing – original draft (equal). **Mengxue Yang:** Data curation (equal); writing – review and editing (equal). **Jian Xu:** Validation (equal); writing – review and editing (equal). **xuebing yan:** Data curation (equal); formal analysis (equal); writing – original draft (lead). **Wenjuan Wu:** Conceptualization (lead); methodology (equal); supervision (equal).

## FUNDING INFORMATION

This research was financially supported by the China National Natural Science Foundation (No.81902422); Program of Jiangsu Commission of Health (No.M2020024); Social Development Program of Yangzhou Science and Technology Bureau (No.YZ2020078); Young Talent supporting program of Jiangsu Science and Technology Association (No.TJ‐2022‐022); Youth Natural Science Foundation of Jiangxi Province (Grant No. 20171BAB215011); and Foundation of Zhaoyang Talent Plan of Shanghai East Hospital (Grant No. DFZY‐6).

## CONFLICT OF INTEREST

The authors declare that they have no known competing financial interests or personal relationships that could have appeared to influence the work reported in this paper.

## ETHICS APPROVAL STATEMENT

This study was approved by the ethics committees of Affiliated Hospital of Yangzhou University (No. 2020‐YKL04‐Y011).

## Supporting information


Figure S1

Figure S2

Figure S3

Figure S4

Table S1

Table S2

Table S3

## Data Availability

The data sets used and/or analyzed in the current study are available from the corresponding author on reasonable request.
