# Supplementary figures and images for: Identification of a novel prognostic signature correlated with epithelial‐mesenchymal transition, N6‐methyladenosine modification, and immune infiltration in colorectal cancer
Source: Cancer Med. 2022 Oct 25;12(5):5926–38. doi: 10.1002/cam4.5384 (PMC10028107; doi:10.1002/cam4.5384)

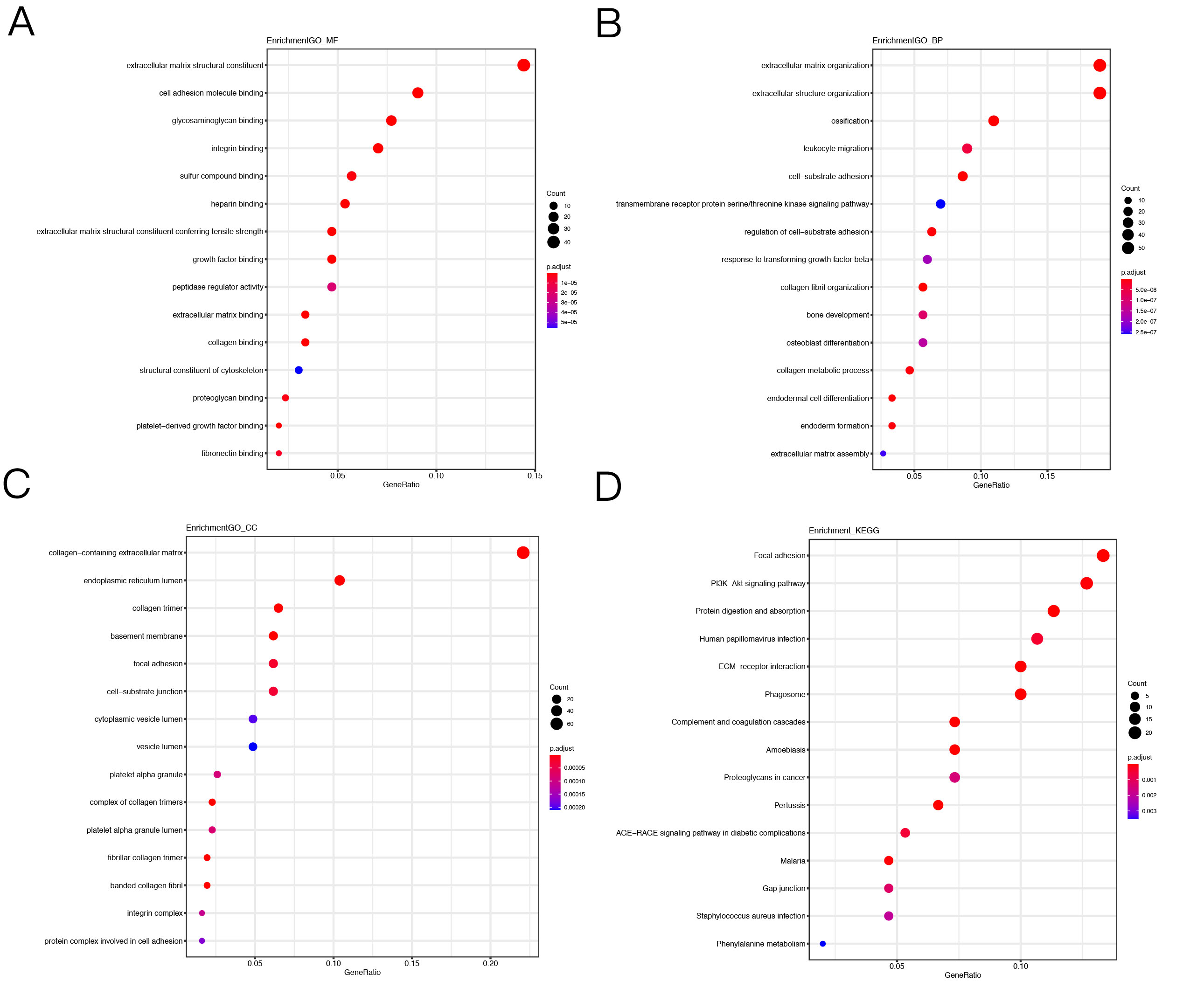

Supplement: Supplementary file 1 — Figure S1 [file CAM4-12-5926-s001.jpg]

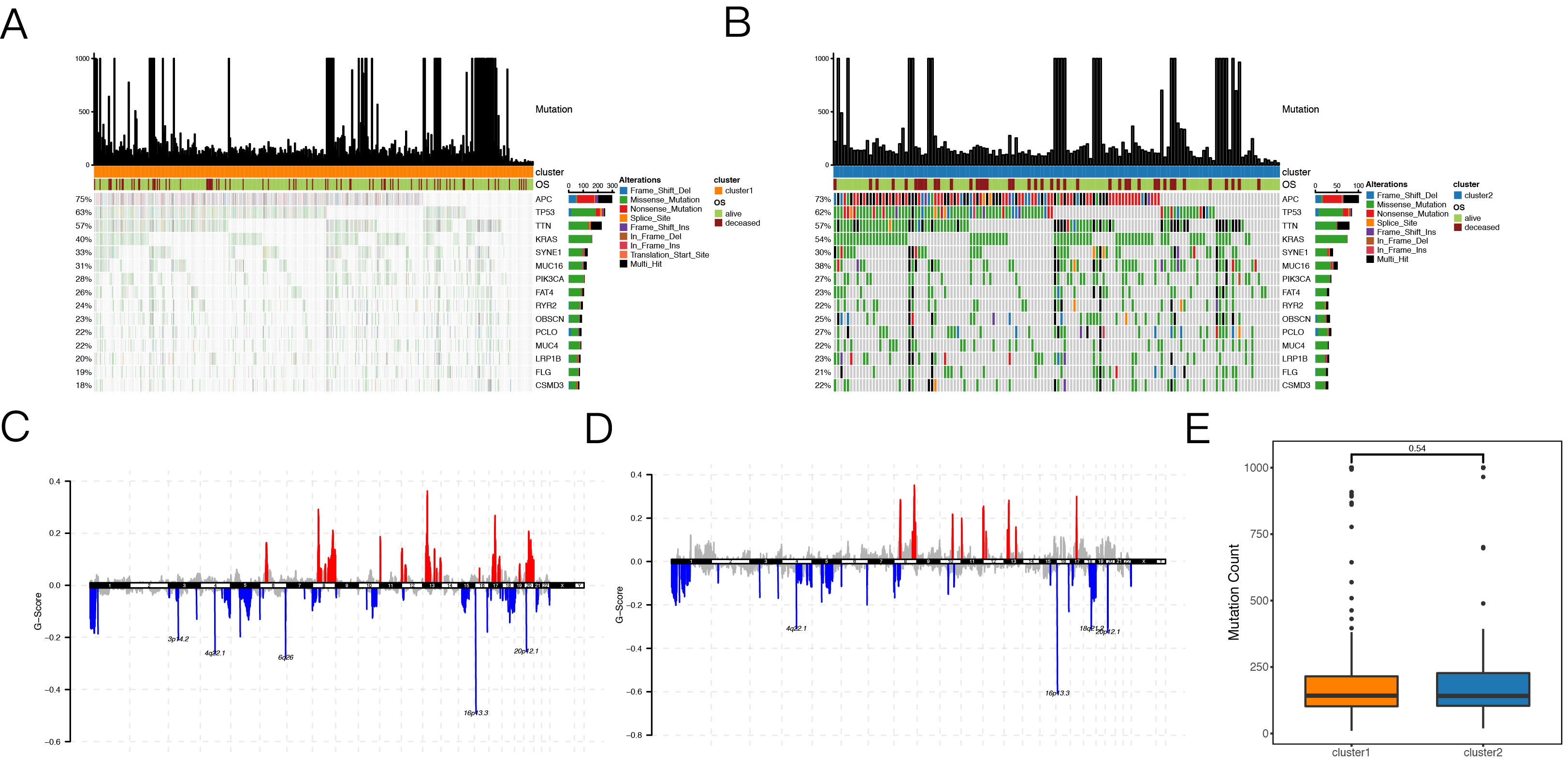

Supplement: Supplementary file 2 — Figure S2 [file CAM4-12-5926-s007.jpg]

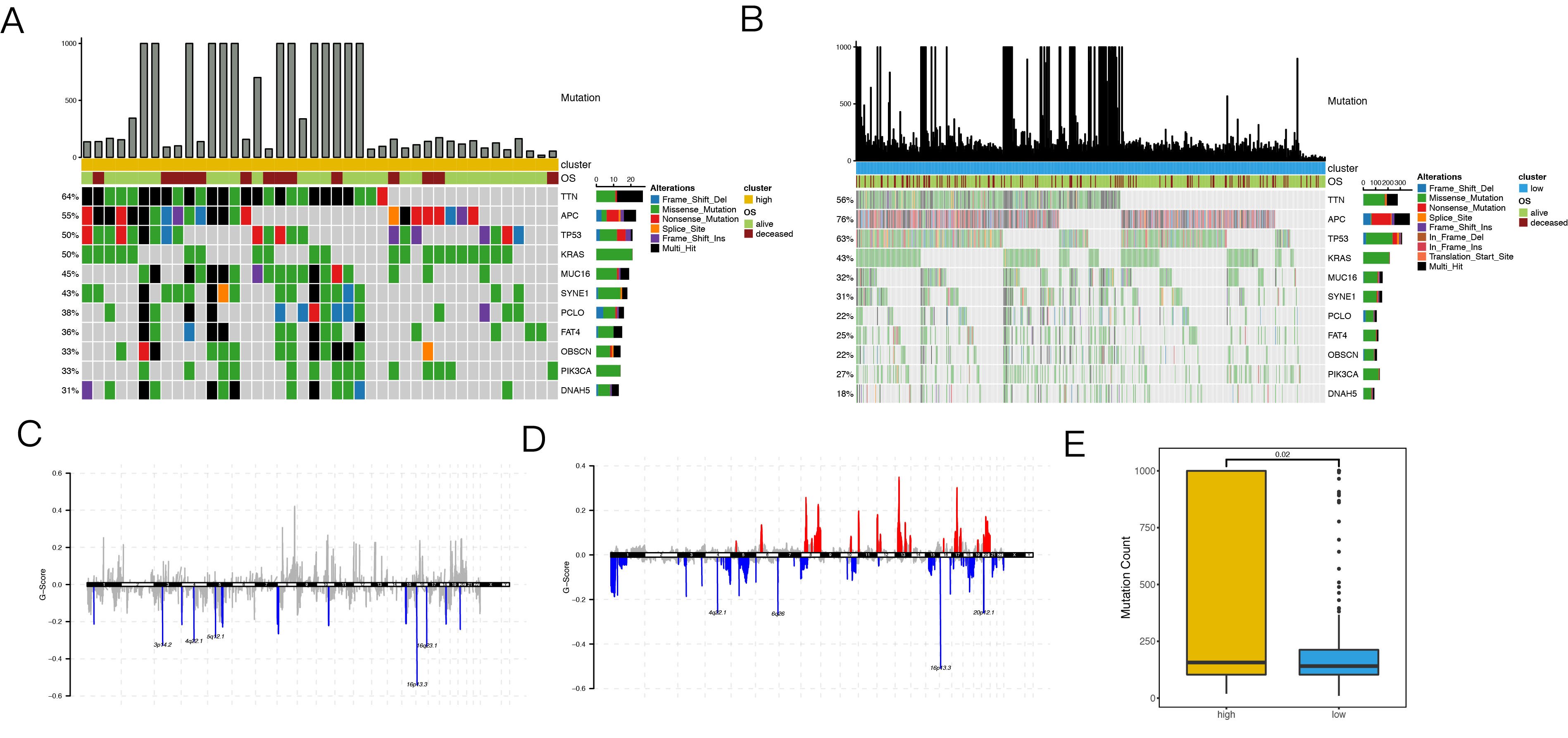

Supplement: Supplementary file 3 — Figure S3 [file CAM4-12-5926-s002.jpg]

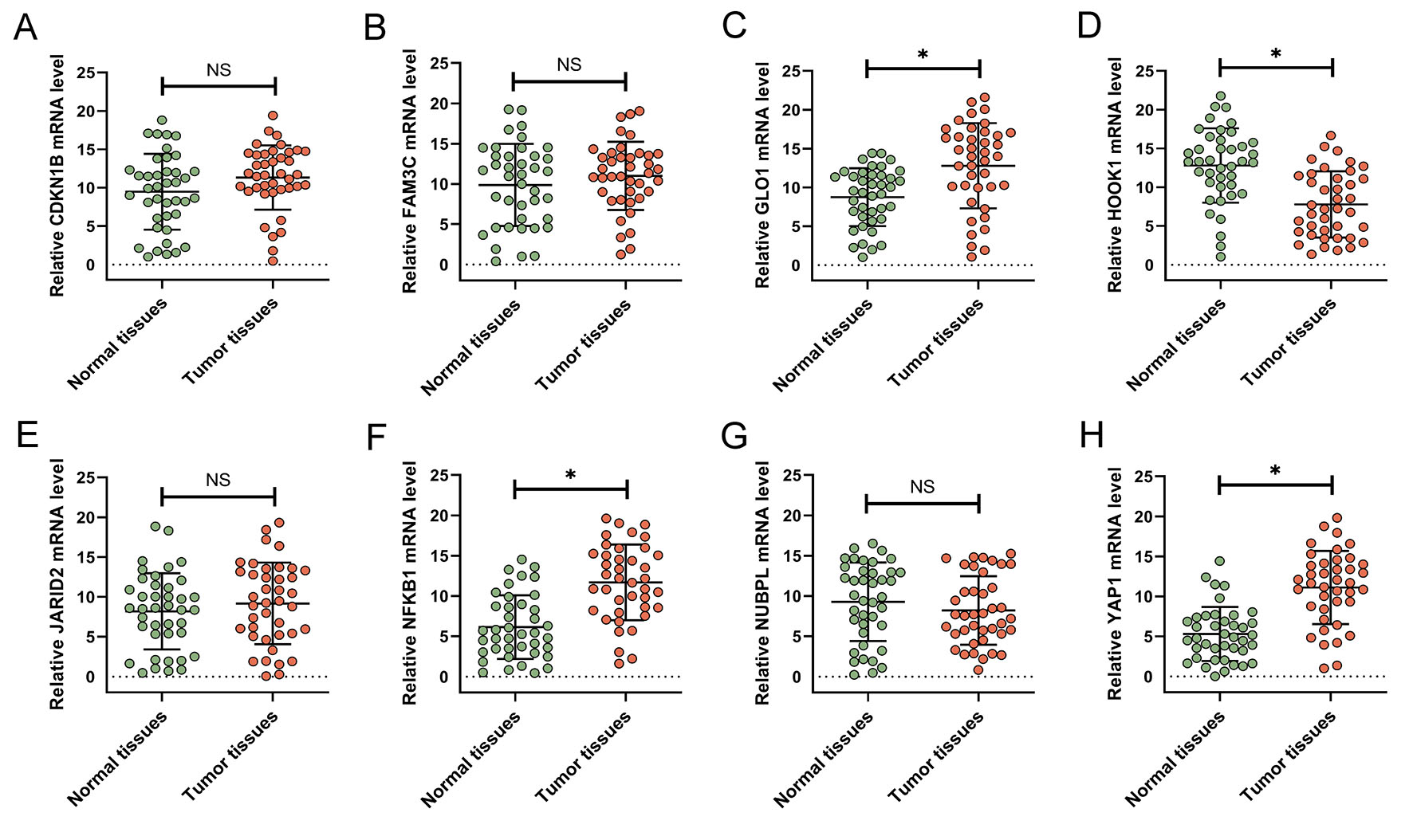

Supplement: Supplementary file 4 — Figure S4 [file CAM4-12-5926-s005.jpg]
