# Supplementary material for: Identification of a novel prognostic signature correlated with epithelial‐mesenchymal transition, N6‐methyladenosine modification, and immune infiltration in colorectal cancer
Source: Cancer Med. 2022 Oct 25;12(5):5926–38. doi: 10.1002/cam4.5384 (PMC10028107; doi:10.1002/cam4.5384)
Supplement: Supplementary file 5 — Table S1 [file CAM4-12-5926-s006.docx]

| Supplementary Table 1. Key molecular alterations of colorectal cancer cell lines | | | | | | | | | | | | | |
| --- | --- | --- | --- | --- | --- | --- | --- | --- | --- | --- | --- | --- | --- |
| Cell lines | c-myc | N-myc | K-ras | H-ras | N-ras | Myb | sis | fos | keratin | CSAp | colon antigen 3 | Mutation | p53 |
| SW620 | + | ND | + | + | + | + | + | + | + | - | - | codon 273 of the p53 gene | NA |
| SW480 | + | ND | + | + | + | + | + | + | + | - | - | codon 12 of the ras proto-oncogene/codon 273 of the p53 gene | + |
| HT-29 | + | ND | + | + | + | + | + | + | NA | NA | NA | codon 273 of the p53 gene | NA |
| LoVo | + | ND | + | + | + | + | + | + | NA | - | - | NA | NA |
| HCT-116 | NA | NA | NA | NA | NA | NA | NA | NA | + | NA | NA | codon 13 of the ras proto-oncogene | NA |

Abbreviation: NA, Not available. ND, Not detected.
