# Supplementary material for: Identification of a novel prognostic signature correlated with epithelial‐mesenchymal transition, N6‐methyladenosine modification, and immune infiltration in colorectal cancer
Source: Cancer Med. 2022 Oct 25;12(5):5926–38. doi: 10.1002/cam4.5384 (PMC10028107; doi:10.1002/cam4.5384)
Supplement: Supplementary file 6 — Table S2 [file CAM4-12-5926-s004.docx]

**Supplementary Table 2.** Primer sequences for the quantitative reverse transcription polymerase chain reaction

| Gene | Orientation | Sequence (5'‑3') |
| --- | --- | --- |
| NUBPL | Forward | CTGAGATGTTTCGCAGAGTCC |
|  | Reverse | CAAGGGTCTGTGCTAGTTTCC |
| FAM3C | Forward | CTTGCCCTGAGAAGCATTTTGC |
|  | Reverse | TCCATCTTGTATGGCCTTCAGAA |
| JARID2 | Forward | ACCAGTCTAAGGGATTAGGACC |
|  | Reverse | TGCTGGGACTATTCGGCTGA |
| HOOK1 | Forward | CAGACATTCAATACTGCCTCACC |
|  | Reverse | CCCCAACATCCTCTTTAATTCGG |
| GIPC2 | Forward | GGCGCGTTTGAAATCTCGC |
|  | Reverse | GTGAGACCAAGTGAATCCTCAG |
| YAP1 | Forward | TAGCCCTGCGTAGCCAGTTA |
|  | Reverse | TCATGCTTAGTCCACTGTCTGT |
| NFKB1 | Forward | AACAGAGAGGATTTCGTTTCCG |
|  | Reverse | TTTGACCTGAGGGTAAGACTTCT |
| GLO1 | Forward | AGCAGACCATGCTACGAGTGA |
|  | Reverse | GAGAGCGCCCAGGCTATTT |
| CDKN1B | Forward | AACGTGCGAGTGTCTAACGG |
|  | Reverse | CCCTCTAGGGGTTTGTGATTCT |
| GAPDH | Forward | GAGAAGGCCTGGGGCTCATTT |
|  | Reverse | AGTGATGGCATGGACTGTGG |
