# Supplementary material for: Identification of a novel prognostic signature correlated with epithelial‐mesenchymal transition, N6‐methyladenosine modification, and immune infiltration in colorectal cancer
Source: Cancer Med. 2022 Oct 25;12(5):5926–38. doi: 10.1002/cam4.5384 (PMC10028107; doi:10.1002/cam4.5384)
Supplement: Supplementary file 7 — Table S3 [file CAM4-12-5926-s003.docx]

Supplementary Table 3. Experimental conditions for the quantitative reverse transcription polymerase chain reaction

| Procedure | Cycle | Temperature | Time |
| --- | --- | --- | --- |
| Stage 1 | 1 | 95℃ | 30 sec |
| Stage 2 | 40 | 95℃ | 3sec |
|  |  | 60℃ | 30 sec |
| Stage 3 | 1 | 95℃ | 15 sec |
|  |  | 60℃ | 60 sec |
|  |  | 95℃ | 15 sec |
